# Oncologic Emergencies: Immune-Based Cancer Therapies and Complications

**DOI:** 10.5811/westjem.2020.1.45898

**Published:** 2020-04-13

**Authors:** Brit Long, Elizabeth Brém, Alex Koyfman

**Affiliations:** * Brooke Army Medical Center, Department of Emergency Medicine, Fort Sam Houston, Texas; † University of California, Irvine Health, Division of Hematology/Oncology, Orange, California; ‡ The University of Texas Southwestern Medical Center, Department of Emergency Medicine, Dallas, Texas

## Abstract

Cancer therapies have undergone several recent advancements. Current cancer treatments include immune-based therapies comprised of checkpoint inhibitors, and adoptive immunotherapy; each treatment has the potential for complications that differ from chemotherapy and radiation. This review evaluates immune-based therapies and their complications for emergency clinicians. Therapy complications include immune-related adverse events (irAE), cytokine release syndrome (CRS), autoimmune toxicity, and chimeric antigen receptor (CAR) T-cell-related encephalopathy syndrome (CRES). Immune-related adverse events are most commonly encountered with checkpoint inhibitors and include dermatologic complications, pneumonitis, colitis/diarrhea, hepatitis, and endocrinopathies. Less common irAEs include nephritis, myocardial injury, neurologic toxicity, ocular diseases, and musculoskeletal complications. CRS and CRES are more commonly associated with CAR T-cell therapy. CRS commonly presents with flu-like illness and symptoms resembling sepsis, but severe myocardial and pulmonary disease may occur. Critically ill patients require resuscitation, broad-spectrum antibiotics, and hematology/oncology consultation.

## INTRODUCTION

Emergency clinicians manage a wide variety of complications associated with malignancy, including cardiovascular, gastrointestinal (GI), pulmonary, infectious, and other complications. Cancer therapies have expanded and improved over the last decade. Immune-based therapies function through a different set of mechanisms compared to prior therapies; thus, this class is associated with different complications.^1^–^4^ Medications and new therapeutic techniques are being continually introduced, and emergency clinicians must understand these medications and their complications.

## METHODS

This is a narrative review evaluating complications from current immune-based therapies in cancer. To complete this review on immune-based therapy complications, we undertook a literature search of PubMed, Google Scholar, and MEDLINE using search terms “immunotherapy,” “immune-based,” “checkpoint inhibitor,” “CAR T,” AND “malignancy” OR “cancer.” We included guidelines, randomized controlled trials, cohort/observational studies, narrative reviews, and systematic reviews/meta-analyses. Studies were limited to English and adult patients. Our initial literature search revealed over 620 resources. We excluded studies not focusing on emergency department (ED) evaluation and management, resulting in inclusion of 134 resources.

## DISCUSSION

### Besides Chemotherapy and Radiation, What Are Other Types of Cancer Therapies?

Immune-based therapies differ from cytotoxic chemotherapy in that immunotherapy works to break the body’s tolerance of the malignant cells. There are several immune-based strategies, each of which acts with different mechanisms (Table 1).^1^–^3^ These treatments can be used in isolation or in combination with chemotherapy and/or radiation.^2^–^6^ However, these therapies can result in either autoimmune or cytokine-associated toxicities that are not seen with chemotherapy and radiation.

Interleukin-2 (IL-2) stimulates the growth of T-cells and natural killer cells, which engage malignant cells and target them for destruction by the immune system. IL-2 was first identified in 1980 and approved in the 1990s for metastatic melanoma and metastatic renal cell cancer, and it currently is used for non-small cell lung cancer as well.^1^,^7^–^9^ IL-2 has demonstrated efficacy in inducing regression in advanced solid cancers.^7^–^9^ While patient responses to therapy can be dramatic when they occur, the true response rate is quite low (objective response rate 15% and five-year overall survival 8% in one European series).^10^ Administration of high-dose IL-2 is done in the inpatient setting as it creates a systemic inflammatory response, often requiring fluids, vasopressors, and intensive monitoring. Few centers administer this therapy. Given the low rate of sustained remissions and high toxicity, the role of high dose IL-2 has substantially decreased with the development of checkpoint inhibitors.

### Checkpoint Inhibitors

**Figure 1:**
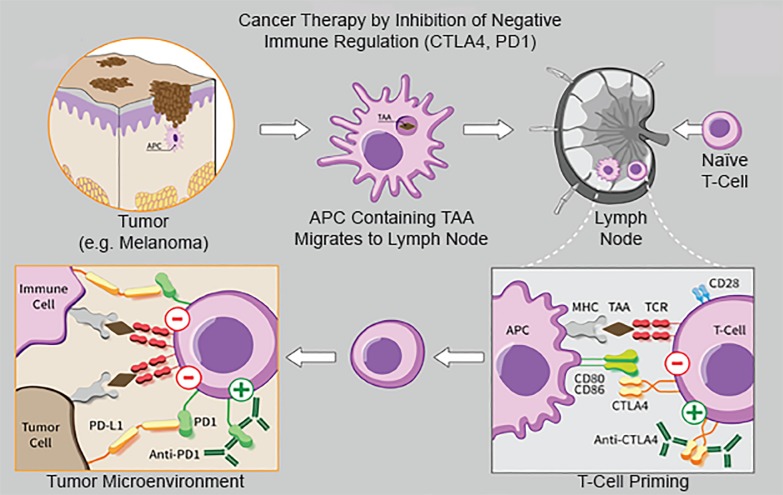
Checkpoint inhibitor mechanisms (CTLA-4 and PD-1). Modified from https://commons.wikimedia.org/wiki/File:11_Hegasy_CTLA4_PD1_Immuntherapie.png. Accessed April 7, 2019. *CTLA4*, cytotoxic T-lymphocyte antigen 4; *PD-1*, programmed cell death protein 1; *PD-L1*, programmed death-ligand 1; *APC*, antigen-presenting cell; *TAA*, tumor-associated antigen; *TCR*, t-cell receptor; *CD*, cluster of differentiation.

Checkpoint inhibitors are monoclonal antibodies that impede the activity of inhibitory molecules on the surface of tumor cells that typically reduce immune response; they take the “brakes” off the immune system so that tumor cells can be recognized.^2^,^11^ Clinical antibodies are available for three ligands: cytotoxic T-lymphocyte antigen 4 (CTLA-4), programmed death 1 (PD-1), and programmed death ligand 1 (PDL-1) (Figure 1). In the absence of malignancy, receptors CTLA-4, PD-1/PD-2, and PDL-1 reduce T-cell proinflammatory response and decrease tissue damage from the immune system, improving self-tolerance.^2^,^11^–^13^ When cancers develop, malignant cells upregulate these receptors, which decreases immune system clearance of these cells.

Checkpoint inhibitors activate T cells by blocking the action of these receptors, removing inhibitory signals and resulting in destruction of tumor cells.^14^–^16^ Inhibition of immune checkpoints can lead to T cells affecting nonmalignant cells, resulting in tissue injury and organ dysfunction.^17^,^18^ Checkpoint inhibitors, specifically ipilimumab, were first approved for metastatic melanoma, although the indications for these agents have drastically expanded, including small cell and non-small-cell lung cancer, ovarian cancer, renal cell carcinoma, gastric and colorectal cancer, urothelial cancer, Hodgkin lymphoma, and others.^2^–^4^,^19^,^20^ These agents have demonstrated significant improvements in survival.^4^,^21^,^22^

**Population Health Research Capsule**

What do we already know about this issue?

Current cancer treatments include immune-based therapies, comprised of checkpoint inhibitors and adoptive immunotherapy, each with the potential for complications.

What was the research question?

We evaluate immune-based therapies and their complications for emergency clinicians.

What was the major finding of the study?

Complications include immune-related adverse events, cytokine release syndrome, and CAR-related encephalopathy syndrome.

How does this improve population health?

Knowledge of the complications associated with immune-based therapies can improve emergency providers’ management of these patients.

### Chimeric Antigen Receptor (CAR) Therapies

**Figure 2:**
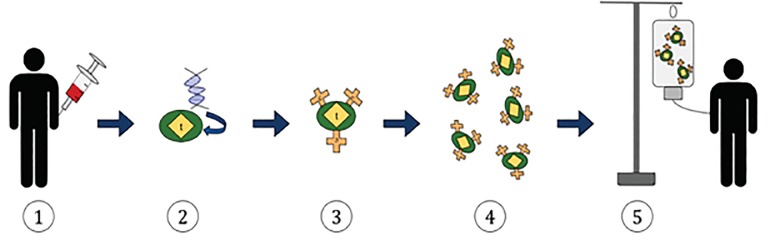
Chimeric antigen receptor (CAR) T-cell therapy process 1) T cells present in the blood are removed from the patient. 2) These T cells are incorporated with the gene-encoding specific antigen receptors. 3) This results in CAR receptors present on the surface of T cells. 4)These modified T cells are harvested and grown in a laboratory setting. 5) The engineered T cells are finally administered to the original patient. Modified from https://commons.wikimedia.org/wiki/File:CAR_T-cell_Therapy.svg. Accessed April 7, 2019.

This method of adoptive immunotherapy uses genetically modified T cells with a chimeric antigen receptor (CAR) that targets malignant cells.^23^–^27^ The patient’s own T cells are obtained through leukapheresis and then modified *ex vivo* with a tumor-specific receptor. The cells with the highest antitumor activity are selected for expansion. Following lymphocyte-depleting chemotherapy, the replicated T cells are then administered to the patient (Figure 2).^23^–^27^ The first CAR T-cell therapies included tisagenlecleucel and axicabtagene ciloleucel, approved in 2017 for lymphoblastic leukemia and advanced lymphoma.^23^–^34^ The Food and Drug Administration-approved CAR T-cell therapies target CD19, a protein expressed on the surface of both malignant and normal B lymphocytes. CAR T-cells targeting a range of other proteins are currently under study for Hodgkin lymphoma, multiple myeloma, glioblastoma, melanoma, breast cancer, and sarcoma.^23^–^34^ Also under development are “natural killer” cells engineered in a similar way to recognize tumor cell antigens.

### What Can Go Wrong with These Therapies?

As the mechanisms of these newer therapies, particularly checkpoint inhibitors and CAR T-cell therapy, significantly differ from normal chemotherapy, adverse effects and complications also differ.^35^–^40^ These complications are typically termed irAEs, which are a result of immune system over-activity, rather than a depleted immune system that occurs with chemotherapy. Immune-related adverse events most commonly affect systems with significant cell turnover.^35^–^40^ Most irAEs occur within 3–6 months of starting therapy, but it should be noted that irAEs can occur at any time, even after the patient discontinues treatment.^35^–^42^ Of patients receiving an anti-CTLA-4 medication, 60–90% experience an irAE, while 39–70% of those administered an anti-PD-1/PD-L1 medication experience an irAE.^5^,^43^–^46^

While mortality is rare, morbidity associated with these agents can be severe.^35^–^42^ Immune-related adverse events associated with this class range in severity, based on a scale from the National Cancer Institute (NCI).^47^ This scale ranges from mild (1) to death (5), based on the Common Terminology Criteria for Adverse Events (CTCAE). Grades 1 and 2, or mild to moderate irAEs, occur frequently and can be treated symptomatically as outpatients. Grade 3 and 4 irAEs, while less frequent, can be severe and require admission (Table 2).^47^ The risk of irAEs and severity is greater with combination therapy, compared to monotherapy.^48^–^51^ Higher doses of ipilimumab and pembrolizumab are also associated with greater risk of irAEs.^52^–^54^ However, other anti-PD-1 and PD-LI medications do not demonstrate a dose-related response with irAE.^19^,^35^–^40^ Interestingly, the occurrence of irAEs is associated with improved clinical outcomes in patients with malignancy.^18^

The most commonly encountered irAEs affect the GI, dermatologic, endocrine, and pulmonary systems. The cardiovascular, hematologic, renal, neurologic, and musculoskeletal systems are not as commonly affected.^35^–^42^ Colitis is associated with better prognosis compared to pneumonitis.^55^,^56^ Dermatologic irAEs are typically seen two to three weeks after therapy initiation, followed by the GI system at six to seven weeks, and the endocrine system at nine to ten weeks.^35^–^42^,^56^ Severe irAEs associated with anti-CTLA-4 medications occur earlier compared to anti-PD-1/PD-L1 medications.^35^–^45^ Laboratory and imaging assessment depend on the specific organ involved. Management focuses on systemic corticosteroids for the majority, except several endocrinopathies (Table 2).^35^–^42^,^48^,^49^ Clinicians should assess for infection and progression of the malignancy, which can present with similar symptoms as an irAE. With appropriate therapy, most irAEs, even grade 3–4, will resolve, except endocrinopathies.^35^–^42^,^48^,^49^

### Dermatologic

Dermatologic toxicities are some of the most common irAEs, especially in patients with melanoma, and are often seen early after starting therapy (two to three weeks).^35^–^37^,^48^,^54^ Reactions may include maculopapular rash, bullae, maculopustular rash, vitiligo, Stevens-Johnson syndrome (SJS), toxic epidermal necrolysis (TEN), or drug reaction with eosinophilia and systemic symptoms. The differential includes vasculitis, atopic/contact dermatitis, viral exanthem, drug toxicity, erythema multiforme, or infectious causes.^35^–^45^,^55^–^58^ Physicians should evaluate the patient’s medication list, hemodynamics, systemic symptoms, mucosal involvement, and total body surface area involved. Laboratory assessment for patients with severe disease includes complete blood count (CBC) with differential, renal and liver function, coagulation panel, creatine kinase (CK), and electrolytes. Dermatology consultation is recommended. Most patients have grade 1–2 rashes. Mild rashes can be treated with oral antihistamines and topical steroids, but for those with severe rashes (including SJS or TEN), systemic steroids are recommended.^55^–^58^

### Cardiac

A variety of cardiac effects can occur including dysrhythmias (blocks, supraventricular or ventricular tachycardias), myocarditis (new systolic heart failure and cardiogenic shock), Takotsubo cardiomyopathy, and pericarditis/myopericarditis.^1^–^4^,^59^,^60^ Patients should be evaluated with electrocardiogram, echocardiography (assessing ventricular function, wall abnormalities, cardiac effusion), thyroid stimulating hormone (TSH), troponin, and chest radiograph. Patients may require cardiac catheterization. Corticosteroid therapy is recommended for myocarditis and ventricular dysrhythmias due to irAE. Prednisone 1–2 milligrams per kilogram per day (mg/kg/day) (or its equivalent) for mild-moderate disease is recommended, but in severe cardiac irAE or in patients who do not respond, methylprednisolone 1 gram intravenous (IV) per day is recommended.^60^–^62^ Other immunologic agents such as infliximab, mycophenolate mofetil, or antithymocyte globulin may be required.^60^–^62^ Dysrhythmias from cardiac conduction pathology may require pacemaker insertion. Heart failure should be treated with standard therapies. Pericardial tamponade requires IV fluid resuscitation and drainage.

### Pulmonary

Pneumonitis is the predominant irAE of the pulmonary system, ranging from no or mild symptoms to respiratory failure requiring intubation and ventilatory support.^1^–^5^ Pneumonitis is the most common irAE requiring discontinuation of checkpoint inhibitor therapy, as well as the most common cause of death related to irAE.^63^–^65^ Dyspnea, cough, fever, and chest pain may be present, with dyspnea occurring in 53% and cough in 35% of patients.^40^,^63^–^65^ Productive cough is rare and suggests another diagnosis.^40^ The differential includes infection (opportunistic infection, pneumonia), heart failure (myocarditis), pulmonary embolism, extension of the malignancy, underlying interstitial lung disease or obstructive disease, diffuse alveolar hemorrhage, neuromuscular disease, or pneumonitis due to other therapies.

Laboratory assessment includes CBC, electrolytes, blood cultures, and urine testing for pneumococcal and legionella antigens. Sputum cultures and gram stain are also recommended, although these can be obtained in the critical care unit.^40^,^63^–^65^ If the patient presents during influenza season, testing for viral upper respiratory infections is recommended. Neuromuscular weakness should be assessed with negative inspiratory force and forced vital capacity.^65^,^66^ Regarding imaging, chest radiograph demonstrates a sensitivity of approximately 75% for diagnosis of pneumonitis.^63^–^66^ Computed tomography (CT) is recommended with contrast. The findings on CT vary, including ground glass opacities (37%), cryptogenic organizing pneumonia, interstitial infiltrates, and pneumonitis not otherwise specified.^65^,^66^

If an infiltrate is present but the patient is asymptomatic, the patient does not require therapy beyond discontinuing the checkpoint inhibitor and obtaining oncology follow-up. Patients with critical illness or grade 3–4 irAEs require other therapies.^63^–^65^ In the patient with severe respiratory symptoms, empiric therapy with antimicrobials is recommended, as grade 3–4 pneumonitis will not be immediately diagnosable. Procalcitonin can be used in the critical care setting to determine whether antibiotics should be continued, but this laboratory assessment should not be used in isolation for determining need for initial antibiotics.^1^–^5^ *Pneumocystis jirovecii* (PJP) can cause similar clinical and radiographic findings, and empiric treatment with trimethoprim-sulfamethoxazole is recommended if the patient is at high risk with corresponding radiographic findings.^63^–^65^ Antifungal therapy is reasonable in the intensive care unit (ICU) if patients do not improve despite use of other therapies. Regarding pneumonitis, 1–2 mg/kg/day of prednisone or methylprednisolone is recommended. Over 80% of patients will improve in several days if an irAE is present.^67^,^68^ However, up to 14% of patients will not respond to corticosteroid therapy.^37^,^67^ Higher doses can be attempted (4 mg/kg/day), as well as other agents including infliximab or mycophenylate.^37^

Patients with grades 3–4 pneumonitis or critical illness will require ICU admission. Bronchoscopy may be needed; however, there is no specific finding on bronchoscopy that definitely diagnoses pneumonitis due to checkpoint inhibitor toxicity, and it is not associated with improved survival.^56^,^57^,^66^–^68^ Bronchoscopy can be used to evaluate for infection as the etiology.^56^,^57^ The decision for bronchoscopy is ultimately left to the pulmonologist and critical care physician.

### Gastrointestinal Presentations

GI presentations of irAE typically include diarrhea and colitis, which can occur in up to 40% on ipilimumab.^1^–^4^ These most often occur 6–7 weeks after initiation of therapy.^1^–^4^,^40^ In the ED, differentiating mild-moderate symptoms from severe diarrhea and colitis is vital, as well as considering the wide differential including infectious diarrhea, enteritis, inflammatory bowel disease, and perforation. Nausea and vomiting may be present with upper GI tract involvement. Colitis tends to present with pancolitis, diverticulosis and segmental colitis, or isolated rectosigmoid colitis but no diverticulosis.^70^ Severe colitis with fever, peritonitis, and perforation is rare but may occur.^57^,^58^,^71^ Stool testing is recommended based on the grade of severity to include bacterial pathogens, viral etiologies, and *Clostridium difficile*.^57^,^58^,^70^,^71^ Imaging with CT of the abdomen/pelvis is recommended in patients with critical illness to evaluate disease severity and complications and may demonstrate thickening of the bowel wall, mesenteric fullness, stranding, and/or perforation.^70^–^73^ Colonoscopy for patients with severe illness may be used to evaluate for alternative diagnoses and guide management. Upper endoscopy may be needed during admission if CT does not reveal findings of colitis but the patient has diarrhea.^72^,^73^

Volume-depleted patients require fluid resuscitation. Opioids and medications such as loperamide should be avoided, if possible. Corticosteroids are recommended for patients with ≥ 6 stools, patients who are admitted for colitis, and other complications, with prednisone at doses of 1–2 mg/kg/day.^37^,^57^,^58^,^73^,^74^ Patients with severe disease may require infliximab.^37^,^57^ Patients with perforation or toxic megacolon require surgical consultation. Empiric antibiotics are recommended for those with severe illness.

### Hepatitis

Patients with hepatitis due to an irAE are usually asymptomatic and detected by routine liver function assessments, although patients may present with fever, jaundice, and abdominal pain.^37^,^75^ Immune-related adverse events affecting the liver typically occur 6–14 weeks after beginning therapy.^35^–^37^,^75^ Liver function tests including aspartate transaminase and alanine aminotransferase, as well as bilirubin, are increased, and the symptoms and levels of elevation determine the severity.^37^,^75^ Fulminant hepatic failure and death are rare. Evaluating for other conditions is recommended, such as alcoholic hepatitis, acetaminophen toxicity, viral hepatitis, chronic hepatitis reactivation, biliary obstruction, shock liver, liver metastases, or vascular occlusion. Laboratory assessment should include viral hepatitis panel and autoimmune hepatitis panel (anti-smooth muscle antibodies, antinuclear antibody, liver-kidney microsomal antibody). Ultrasound of the liver, gallbladder, and biliary tract with doppler examination is needed to evaluate for liver and/or biliary disease.^37^,^75^ Treatment includes stopping medications that may result in hepatoxicity. Those with grade 2 disease or higher should receive corticosteroids. Infliximab may result in hepatotoxicity, limiting its use in hepatitis due to irAE.^2^,^35^–^37^

### Nephritis

Acute tubulointerstitial nephritis is the most common form of renal toxicity, although glomerulonephritis can also occur.^2^,^35^–^40^ Patients are typically asymptomatic, with laboratory abnormality the only finding. Renal failure can present with uremic encephalopathy, volume overload, and electrolyte abnormalities.^2^,^76^,^77^ As renal injury due to irAE is rare, evaluation for other causes of renal failure is recommended to include CBC and blood smear (microangiopathic hemolytic anemias), CK (myositis and rhabdomyolysis), and urinalysis are recommended. Urinalysis may be normal or include sterile pyuria, mild proteinuria, microscopic hematuria, and granular casts.^76^,^77^ Renal ultrasound is recommended with vascular studies. If no other cause is found for renal injury, biopsy is recommended. Corticosteroids should be initiated if the renal injury is due to irAE. Other immunosuppressant medications may be needed if the patient does not improve. Hemodialysis is recommended for grade 4 irAEs.^2^,^35^–^40^,^76^,^77^

### Endocrinopathies

Endocrinopathy affects approximately 10% of patients; it can be either central involving the pituitary gland, or peripheral involving the thyroid or adrenal glands.^2^,^48^ Immune-related adverse events affecting the endocrine system are difficult to diagnose, and clinicians should consider these conditions in patients with non-specific symptoms such as fatigue, weakness, headache, nausea, and vomiting.^2^,^40^,^48^,^54^ These complications typically arise 9–10 weeks after initiation of therapy. Endocrinopathies differ from other irAEs with organ dysfunction, as treatment with corticosteroids is not typically used, and organ dysfunction is often persistent due to disruption of the adrenal axis.^2^,^35^–^40^,^61^ Thus, treatment typically involves hormone replacement on a long-term basis.

Primary hypothyroidism is the most common endocrinopathy and may present with fatigue, cold intolerance, weight gain, constipation, and depression.^2^,^37^,^48^,^78^ Diagnosis involves elevated TSH and low free T4, and treatment includes thyroid hormone replacement. Hyperthyroidism comes in two forms: thyroiditis and Graves’ disease. Symptoms of hyperthyroidism include heat intolerance, diaphoresis, dyspnea, diarrhea, palpitations, tremor, and weight loss, although Graves’ disease can present with ophthalmopathy.^2^,^40^,^48^,^79^ Thyroiditis presents with mild symptoms of hyperthyroidism in the acute phase, followed by chronic hypothyroidism due to gland destruction. Graves’ disease is much less common and presents with persistent, more severe hyperthyroidism.^2^,^48^,^79^ Diagnosis includes reduced TSH and elevated free T4. TSH-receptor antibody can be used as well. Treatment includes a thionamide and endocrinology consult for those with mild-moderate symptoms.^2^,^37^,^40^ Severe symptoms require therapy for thyrotoxicosis and thyroid storm if present, as well as corticosteroids.^37^

Adrenal insufficiency can present with fatigue and weight loss or with adrenal crisis and distributive shock. Electrolytes, renal and liver function, cortisol, adrenocorticotropic hormone (ACTH), and CT of the abdomen/pelvis are recommended. Treatment includes hydrocortisone, with dosing dependent on patient hemodynamic status and symptoms. Adrenal crisis requires hydrocortisone 100 mg IV, as well as evaluation for infection, broad-spectrum antibiotics, and IV fluids with glucose.^2^,^35^–^40^,^48^

Pituitary dysfunction, or hypophysitis, typically presents with symptoms of hypothyroidism but may also present with arthralgias, vision changes, hypogonadism, hypothyroidism, diabetes insipidus, and/or adrenal insufficiency.^2^,^35^–^40^,^48^,^54^,^79^ Headache occurs in 85% of patients.^40^ Electrolytes, cortisol, ACTH, TSH, free T4, luteinizing hormone, follicle-stimulating hormone, and either testosterone/estrogen level are recommended, as well as central nervous imaging with brain MRI.^37^–^40^ Diagnosis is based on at least one hormone deficiency plus MRI abnormality, or ≥ 2 hormone deficiencies with headache.^37^–^40^ Treatment is based on patient presentation, symptoms, and laboratory results.^37^,^40^ Insulin-dependent diabetes is another irAE.^2^,^37^,^80^ Treatment focuses on insulin and management of diabetic ketoacidosis if present.^37^

### Neurologic

Neurologic irAEs are rare (1–6%) but may include myasthenia gravis with fatigable and fluctuating weakness primarily affecting ocular and bulbar muscles.^40^,^81^–^86^ Conditions that may present in a similar manner include myositis, spinal cord pathology, and Guillain-Barré syndrome (Miller-Fisher variant).^81^–^85^ Laboratory assessment includes CK, electrolytes, and MRI of the brain and/or spine dependent on the patient presentation.^81^–^86^ Admitted patients should be tested for acetylcholine receptor and anti-striated muscle antibodies, as well as electrodiagnostic studies. Treatment includes prednisone 1–1.5 mg/kg/day and pyridostigmine. Intravenous immunoglobulin (IVIG) or plasmapheresis for severe symptoms is recommended.^81^–^86^ Medications such as beta blockers, magnesium IV, fluoroquinolones, macrolides, and other medications that can worsen myasthenia must be avoided.

Guillain-Barré syndrome can present with progressive, ascending muscle weakness, often beginning with neuropathic pain and/or sensory changes in the lower extremities.^2^,^37^,^81^,^83^,^87^ Reflexes are typically absent. This disease may cause respiratory failure with respiratory muscle involvement and dysautonomia. CK, spine MRI, lumbar puncture (LP) (elevated protein), and electrodiagnostic studies are recommended.^88^–^90^ Treatment includes IVIG or plasmapheresis.^87^–^90^

Transverse myelitis presents with bilateral acute/subacute weakness or sensory changes.^89^,^90^ Reflexes are increased, as opposed to Guillain-Barré syndrome.^40^,^81^–^85^ Assessment with MRI of the spine and LP is recommended, with management including methylprednisolone 2 mg/kg/day or 1 g/day IV.^81^–^85^,^90^

Encephalitis may present with confusion, headache, seizures, focal weakness, or other focal findings such as altered speech.^2^,^37^,^81^,^83^ LP with central nervous system imaging is recommended. LP may reveal lymphocytic pleocytosis and elevated protein, and imaging is often normal, although brain MRI may reveal T2-weighted-fluid-attenuated inversion recovery signals.^89^–^91^ Patients should be treated with antibiotics and acyclovir until infection is excluded.^40^,^81^–^85^ Methylprednisolone 1–2 mg/kg IV is recommended, although patients with the presence of severe symptoms or oligoclonal bands should be managed with methylprednisolone 1 g with IVIG.^81^–^85^

Other neurologic conditions include neuropathies, aseptic meningitis, multiple sclerosis, optic neuritis, and posterior reversible encephalopathy syndrome.^81^–^85^,^90^,^91^

### Hematologic

Checkpoint inhibitors can affect all blood cell lines, resulting in a variety of hematologic abnormalities.^92^–^102^ Anemia can be due do an autoimmune hemolytic type or aplastic.^92^–^97^ Autoimmune hemolytic anemia can present with weakness, jaundice, pallor, dark urine, and fatigue. Evaluation includes CBC with differential, peripheral smear, reticulocyte count, lactate dehydrogenase, haptoglobin, coagulation panel, fibrinogen, and direct agglutinin test.^92^–^96^ Treatment for confirmed hemolytic anemia includes prednisone 1–2 mg/kg/day with folic acid. Transfusion with irradiated and filtered products is recommended if hemoglobin is < 7 milligrams per deciliter (mg/dL).^32^,^92^–^97^

Immune thrombocytopenia presents with petechiae and bleeding.^2^,^37^,^97^,^98^ Laboratory assessment is similar to that in hemolytic anemia, though testing for HIV and hepatitis B/C is recommended. Treatment includes prednisone and IVIG. Bone marrow aspiration may be required during admission.^2^,^37^,^97^,^98^

Lymphopenia may lead to opportunistic infections such as *Pneumocystis jirovecii* pneumonia (PJP), as well as other infections similar to human immunodeficiency virus (HIV).^2^,^3^,^37^,^97^–^100^ Patients should be evaluated for HIV and cytomegalovirus (CMV). Chest radiograph, HIV testing, and CD4 T cell count are recommended.^2^,^3^,^37^,^97^ If patients present with a lymphocyte count < 250 cells per millimeter cubed (cells/mm^3^), then prophylaxis for PJP and mycobacterium avium complex is recommended.

Aplastic anemia presents with findings of anemia, thrombocytopenia, and lymphopenia/neutropenia.^2^,^3^,^97^–^101^ Evaluation for viral diseases such as CMV, Epstein-Barr virus, HIV, and parvovirus is recommended, along with B12/folate levels and bone marrow aspiration during admission. Patients with severe aplastic anemia without a clear secondary cause may require anti-thymocyte globulin and cyclosporine.^2^,^3^,^40^,^97^–^101^

A dangerous hemolytic irAE is acquired thrombotic thrombocytopenic purpura (TTP) or atypical hemolytic uremic syndrome (aHUS).^2^,^3^,^40^,^92^,^97^ Both conditions can present with non-palpable purpura, fever, abdominal pain/vomiting, and renal failure. TTP tends to present with neurologic abnormalities. Lactate dehydrogenase, haptoglobin, coagulation panel, fibrinogen, ADAMTS13 activity and inhibitor titer, complement, and urinalysis are recommended, along with the other common laboratory assessments for anemia. If diarrhea is present, testing for bacterial pathogens is recommended. Treatment depends on the diagnosis. Prednisone or methylprednisolone can be used, but for TTP, plasma exchange is recommended, while for aHUS eculizumab is recommended.^2^,^3^,^40^,^92^,^97^

Acquired hemophilia can occur due to inhibition of factor VIII.^97^,^101^,^102^ Mixing studies and quantification of inhibitor levels are used to make the diagnosis. Treatment includes prednisone and/or other immunosuppressive therapies.^37^,^97^

### Rheumatologic

Myalgias and arthralgias are present in 2–12% of patients, most commonly in those receiving anti-PD-1 agents.^37^,^40^,^91^ However, vasculitis, myositis, and giant cell arteritis may occur. Patients with mild to moderate symptoms can be treated with acetaminophen and/or non-steroidal anti-inflammatory drugs. Prednisone can also be used. Severe symptoms should be treated with high-dose corticosteroids, with consultation with oncology and rheumatology.^2^,^3^,^37^,^40^,^91^

### Ocular

Ocular toxicity is rare, occurring in 1% of patients.^2^,^3^,^103^ These are divided into ocular inflammation including keratitis, uveitis and orbital inflammation, and retinal/choroidal disease. Patients may present with eye pain and vision changes. Treatment requires ophthalmology consultation with topical corticosteroids for episcleritis or anterior uveitis. Systemic corticosteroids are recommended for severe inflammation.^2^,^3^,^103^

### Unique Toxicities of CAR T-Cell Therapy

Regarding CAR T-cell therapy, B cell aplasia is common with use of CD19-directed CAR T-cell therapy, given that CD19 is all expressed on normal mature B cells.^23^–^27^ Hypogammaglobulinemia may be present due to B cell depletion. Other hematologic toxicities can occur such as anemia or thrombocytopenia.^23^–^27^ Major side effects include cytokine release syndrome (CRS) and neurologic effects such as CAR T-cell-related encephalopathy syndrome (CRES).^27^,^104^–^106^

CRS occurs with massive release of cytokines.^104^–^106^ It may affect up to 90% of patients receiving CAR T-cell therapy, with half severe, requiring critical care and vasopressors and/or ventilation.^107^–^110^ Cytokines are proteins that act as signaling among cells and result in systemic inflammation. Interleukin (IL)-1 and IL-6 are central factors in CRS toxicity.^110^–^115^ This may result in a variety of symptoms, ranging from mild flu-like symptoms to hypotension and death (Table 3).^104^–^106^ Symptom onset in CRS varies, depending primarily on the agent and severity of immune cell activation. CRS most commonly occurs one to five days after CAR T-cell infusion, although it may occur weeks later.^104^–^115^ Patients with large tumor burdens may experience more severe symptoms. There is no correlation between CRS and clinical response to therapy.^104^–^106^

As Table 3 demonstrates, signs and symptoms of CRS overlap with sepsis, macrophage activation syndrome (hemophagocytic lymphohistiocytosis), neutropenic fever, and tumor lysis syndrome.^104^–^106^,^116^–^120^ Due to the increased levels of inflammatory markers, patients develop systemic inflammation, beginning with fever, which may reach over 40.0° C.^121^ Fever usually precedes the onset of CRS by at least one day.^104^–^106^,^116^–^120^ Those with fever receiving CAR T-cell therapy should be admitted and monitored for CRS. The severity can range from mild, treated with supportive care, to life-threatening, with a wide variety of signs and symptoms.^104^–^106^,^116^–^120^ Flu-like symptoms such as fatigue, headache, rash, arthralgias, and myalgias are common. Severe cases can include hypotension and shock, disseminated intravascular coagulation, and multiorgan failure.^104^–^106^,^116^–^120^ Respiratory symptoms such as cough and tachypnea can progress to acute respiratory distress syndrome. Cardiac toxicity can be life-threatening and presents in a similar manner to sepsis or stress cardiomyopathy; however, it is often reversible.^104^–^106^,^122^,^123^

The NCI has developed a grading system for severity (Table 4).^104^–^106^ Laboratory results often reveal elevated liver function tests and bilirubin, increased or decreased white blood cells and platelets, low fibrinogen, elevated blood urea nitrogen (BUN), and increased D-dimer. Chest radiograph, urinalysis, and blood cultures are recommended due to the high risk of infection.^104^–^106^,^118^ C-reactive protein (CRP) and erythrocyte sedimentation rate (ESR) may be elevated, but normal results cannot exclude CRS. CRP is often elevated in CRS, as it is associated with IL-6 production^117^,^124^–^127^ However, CRP cannot differentiate between CRS and infection.^124^–^127^ While not typically obtained in the ED, ferritin is often elevated in CRS, similar to that seen in macrophage activation syndrome.^104^–^106^,^128^

Management of CRS requires symptomatic care and cytokine inhibition. Depending on signs, symptoms, and patient hemodynamic status, patients may require IV fluids, vasopressors, and broad-spectrum antibiotics, as sepsis is possible (Table 4).^104^–^106^,^117^,^118^ Management of CRS focuses on two medications: corticosteroids and tocilizumab, a humanized immunoglobulin that prevents IL-6 binding to other receptors and further cell signaling.^104^–^106^ Literature suggests tocilizumab is an effective therapy for severe CRS, with improvement within hours of infusion.^129^–^131^ Corticosteroids are generally avoided in CRS and should only be used in conjunction with oncology consultation, as these medications can adversely affect antitumor effects. This differs from checkpoint inhibitor therapy, in which corticosteroids do not affect the therapeutic effects on the malignancy.^117^,^132^,^133^ However, if evidence of adrenal crisis is present, stress-dose corticosteroids are recommended. Patients with CRS require admission and consultation with oncology due to the potential severe nature of the condition.^104^–^106^

Differentiating CRS from infection and sepsis is difficult, as patients will typically meet systemic inflammatory response syndrome criteria and have greater than two points on the sequential organ failure assessment score.^118^,^121^ One study found that 23% of patients developed infection in the first month of CAR T-cell therapy, with many infections occurring with onset of CRS.^118^ Bacterial infections predominate, primarily of the respiratory tract. Thus, patients should be presumed to have infection, and antibiotics and resuscitation are recommended in the ED.^104^–^106^

Cytokine storm is due to nonspecific activation of T cells, usually rapidly after CAR T-cell infusion.^119^,^120^ This condition is a separate entity from CRS, although the presentation is similar. Tumor necrosis factor and interferon gamma are the predominant factors resulting in cytokine storm.^104^–^106^,^119^,^120^ As opposed to CRS, the primary therapy for cytokine storm includes corticosteroids with resuscitation. However, corticosteroids should only be initiated with oncologist consultation, as steroids can deplete CAR T-cells.^104^–^106^

Neurologic complications from CAR T-cell therapy present in a wide range, from mild headache to severe altered mental status and seizures.^104^,^120^ Neurotoxicity is the second most common major adverse event with CAR T-cell therapy, officially known as CRES.^104^–^106^ CRES does not always coincide with CRS, and symptoms can occur before, during, or after CRS.^104^ A mechanism has not been definitely determined, although IL-1 may play a role. Tocilizumab is not effective in CRES as it does not cross the blood–brain barrier; however, anakinra, which blocks IL-1 receptors, may be beneficial.^105^ Patients with severe neurologic symptoms should be managed with corticosteroids, primarily dexamethasone 10 mg IV due to its ability to cross the blood–brain barrier.^104^,^134^

### What’s the Emergency Physician to Do?

While irAEs from checkpoint inhibitors and complications of CAR T-cell therapy can be severe, physicians must consider several other causes of the patient’s symptoms. Patients may be experiencing acute illness unrelated to the malignancy and therapy, complications of the malignancy itself (disease progression or other complication such as tumor lysis syndrome), complications of more traditional cancer therapies including chemotherapy and radiotherapy (radiation pneumonitis, opportunistic infections/neutropenic fever), and complications of the immune-based therapy itself (irAEs, underlying rheumatologic disorder, immune reconstitution syndrome).^1^–^3^,^12^,^35^–^40^ With this wide differential of potentially dangerous conditions, patients may require emergent resuscitation.

Obtaining history of immune-based therapy is vital in the consideration of irAEs, CRS, or CRES. In the ED, biomarkers such as interleukin levels are not readily available. Laboratory assessment should include CBC with differential, renal function and electrolytes, coagulation studies, liver function testing, cortisol, TSH, and urinalysis. Inflammatory markers such as CRP and ESR may be beneficial but cannot definitively diagnose the condition or exclude infection. Suspicion of an infectious etiology requires blood cultures and antibiotics. Bedside echocardiography can assist in assessment of the cardiopulmonary system.^1^–^4^,^12^,^35^–^40^

Management focuses on the specific organ involved, with resuscitation and antibiotics. For severe, critical illness, the checkpoint inhibitor should be discontinued if an irAE is likely. Early initiation of a corticosteroid improves prognosis with irAEs due to checkpoint inhibitors, except for those with endocrinopathies, and most patients improve within 2–3 days. If patients do not improve, there are other immunosuppressive agents that can be used.^1^–^3^,^71^ Recurrence of an irAE can be seen with corticosteroid tapering and checkpoint inhibitor reinitiation.^1^–^3^,^12^,^37^ Regarding CRS related to CAR T-cell therapy, patients should be admitted to the ICU for tocilizumab, and CRES requires ICU admission and corticosteroids.^35^–^40^

## CONCLUSION

Immune-based therapies consist of immune stimulators, checkpoint inhibitors, and adoptive immunotherapy. These therapies differ in mechanism compared to other anticancer therapies, namely chemotherapy and radiation. Complications include irAEs, CRS, autoimmune toxicity, and CRES. Immune-related adverse events, most commonly encountered with checkpoint inhibitors, may result in dermatologic complications, pneumonitis, colitis/diarrhea, hepatitis, and endocrinopathies. Less common irAEs include nephritis, myocardial injury, neurologic toxicity, ocular diseases, and musculoskeletal complications. Cytokine release syndrome and CRES are more commonly associated with CAR T-cell therapy. Cytokine release syndrome may present with flu-like illness, but severe myocardial and pulmonary disease may occur. Critically ill patients require resuscitation, broad-spectrum antibiotics, and hematology/oncology consultation.

## Figures and Tables

**Table 1:** Immune-based agents and mechanisms used in cancer therapy.^1^–^3^

Class	Mechanism	Therapeutic agent
Stimulator	Stimulates effector cells	Interleukin-2
Checkpoint inhibitor	Inhibits regulatory factors	Anti-CTLA4 (ipilimumab), Anti-PD-1 (nivolumab, pembrolizumab), Anti-PD-L1 (atezolizumab, avelumab, durvalumab)
Adoptive immunotherapy	Activated immune cell passive transfer, which have antitumor activity	CAR T-cell (axicabtagene ciloleucel, tisagenlecleucel)

*CAR*, chimeric antigen receptor; *CTLA4*, cytotoxic T-lymphocyte-associated protein 4; *PD-1*, programmed cell death protein 1; *PD-L1*, programmed death-ligand 1.

**Table 2:** Immune-related adverse effects on the organs of cancer patients undergoing immunotherapy.^35^–^42^,^48^,^49^

Organ System	Grade	Definition	Management	Disposition
**Dermatologic** Most common agents: anti-CTLA-4 inhibitors (Ipilimumab), especially melanoma, but also associated with anti-PD-1/PD-L1 therapy	1	- Nonlocalized, diffuse rash, < 10% BSA - Mild pruritis	- Provide oral antihistamines, class I topical corticosteroid (class V/VI for face)	- Discharge with oncology follow- up and dermatology referral - Provide return precautions - If symptoms worsen, treat as grade 3/4
	2	- Maculopapular rash with 10–30% BSA - Intense, widespread rash pruritis, may have excoriations	- Similar to grade 1 - Add systemic corticosteroids (prednisone 0.5–1 mg/kg/day)	- Similar to grade 1 - If symptoms worsen, treat as grade 3/4
	3–4	- Maculopapular rash > 30% BSA - Intense pruritis, limits ADLs, sleep - Stevens-Johnson syndrome or toxic epidermal necrolysis - Full thickness dermal ulceration or necrotic, bullous, or hemorrhagic findings	- Evaluate and exclude systemic hypersensitivity - Obtain serum tests with CBC with differential, complete metabolic panel - Provide systemic corticosteroids - Provide oral antihistamines and GABA agonist (pregabalin or gabapentin)	- Admit with monitoring - Emergent dermatology consult

**Gastrointestinal** Most common agents: anti-CTLA-4 inhibitors (Ipilimumab), but also associated with anti-PD-1/PD-L1 therapy	1	- Diarrhea ≤ 4 stools/day - Asymptomatic colitis	- Observe patient, obtain stool and serum studies - May provide antidiarrheal medications, but no strong recommendations	- Ensure follow up with oncology as outpatient - Provide return precautions - Treat as grade 2 if worsening symptoms
	2	- Diarrhea 4–6 stools/day - Colitis with abdominal pain, blood/mucous in stool	- Observe patient if diarrhea only, obtain serum and stool studies - CRP, ESR, fecal calprotectin, lactoferrin, imaging optional - Antidiarrheal medications not recommended - If diarrhea and colitis present, provide prednisone 1 mg/kg/day	- Obtain follow up with oncology - Provide return precautions - If no improvement in 2 days, increase prednisone to 2 mg/kg/day
	3–4	- Diarrhea > 7 stools/day, incontinence, requiring IV fluids for > 1 day, unable to do ADLs - Colitis with severe pain, ileus, fever - Grade 4 with peritoneal findings	- Admit patient, obtain serum and stool markers, inflammatory markers, imaging, and GI consult - Prednisone 1–2 mg/kg/day - Provide antibiotics - May require other anti-inflammatory medications	- Oncology and GI consult with admission - Infliximab may be needed (do not use in perforation or septic shock)

**Hepatitis** Most common agents: combined anti-CTLA-4 inhibitor plus anti-PD-1/PD-L1 therapy; isolated therapy less commonly associated	1	- Elevated AST, ALT to 3 X ULN - Elevated total bilirubin up to 1.5 X ULN	- Evaluate and exclude infection, drug injury, thrombotic, or malignant causes	- Follow up with oncology or primary provider for repeat examination and testing
	2	- Elevated AST, ALT > 3 X ULN to 5 X ULN - Elevated total bilirubin 1.5 X ULN to 3 X ULN	- Evaluate and exclude infection, drug injury, thrombotic, or malignant causes - Prednisone 0.5–1 mg/kg/day	- Follow up with oncology or primary provider for repeat examination and testing in 1–2 days
	3–4	- Elevated AST, ALT > 5 X ULN - Elevated total bilirubin > 3 X ULN	- Prednisone 1–2 mg/kg/day - Provide antibiotics for opportunistic infections - Consult GI	- Admit patient - If patient does not improve in 3–5 days, other immunosuppressant medications needed

**Pulmonary** Most common agents: combined anti-CTLA-4 inhibitor plus anti-PD-1/PD-L1 therapy; isolated therapy less commonly associated	1	- Asymptomatic	- Monitor symptoms/oxygen saturation - Consider imaging before reinitiating checkpoint inhibitor	- Consider pulmonary, ID consult - Ensure follow up with oncology as outpatient; provide return precautions - If saturation falls < 92%, recommend home pulse oximetry
	2	- Symptoms limiting ADLs, mild/moderate hypoxia	- Systemic corticosteroids (prednisone 1 mg/kg/day) - Consider prophylactic antibiotics - Bronchoscopy or lung biopsy may be required	- Consider pulmonary, ID consult - Discuss with oncology - Admit to observation unit
	3–4	- Severe new symptoms, worsening/severe hypoxia	- Methylprednisolone 2 mg/kg/day IV - Patients with severe symptoms may require infliximab, cyclophosphamide, IVIG, or mycophenolate - Consider prophylactic antibiotics - Bronchoscopy or lung biopsy may be required	- Consult pulmonary and ID specialists - Admit with monitoring, consider ICU care

**Nephritis** Less commonly affected than other systems; most commonly with combined anti-PD-1/PD-L1 and anti-CTLA-4 inhibitor therapy	1	- Asymptomatic - Creatinine increase > 0.3 mg/dL or Creatinine > 1.5–2 X ULN	- No treatment needed - Consider discontinuing checkpoint inhibitor	- Follow up with oncology and obtain outpatient referral
	2	- Creatinine > 2–3 X ULN	- Discontinue checkpoint inhibitor - Evaluate for other etiologies of renal injury with laboratory assessment and ultrasound - Prednisone 0.5–1 mg/kg/day	- Follow up with oncology and obtain nephrology referral
	3–4	- Creatinine > 4 mg/dL or Creatinine > 3 X ULN - Grade 4 marked by life-threatening electrolyte abnormalities	- Discontinue checkpoint inhibitor - Evaluate for other etiologies of renal injury with laboratory assessment and ultrasound - Prednisone 1–2 mg/kg/day - Biopsy typically required	- Admit to hospital - Consult oncology and nephrology specialists

**Endocrine** Most common agents: anti-PD-1/PD-L1 therapy and anti-CTLA-4 inhibitor therapy	1	- Asymptomatic or mild symptoms	- No treatment needed	- Follow up with oncology and obtain outpatient endocrine referral
	2–3	- Evidence of endocrine dysfunctions with weakness, fatigue	- Treat based on condition - Hypophysitis: Obtain TSH, T4, cortisol; MR sella; prednisone 1–2 mg/kg/day if imaging abnormal - Adrenal insufficiency (central): hydrocortisone 100 mg IV - Diabetes (insulin-dependent): Start insulin, evaluate for DKA - Hypothyroidism: Start levothyroxine - Hyperthyroidism: Treat with beta-blockers if symptoms present, treat Graves’ disease if present	- Endocrinology consult - Admit to hospital
	4	- Adrenal crisis may be present (dehydration, shock) - Visual field deficits, severe headache	- Evaluate and exclude sepsis - Manage adrenal crisis (hydrocortisone) - Resuscitate with IV fluids - Treat with prophylactic antibiotics	- Emergent endocrinology consultation - Admit to ICU for further evaluation and monitoring

*CTLA4*, cytotoxic T-lymphocyte antigen 4; *PD-1*, programmed cell death protein 1; *PD-L1*, programmed death-ligand 1; *BSA*, body surface area; *GABA*, gamma-aminobutyric acid; *AST*, aspartate aminotransferase; *ALT*, alanine aminotransferase; *ADLs*, activities of daily living; *mg*, milligram; *kg*, kilogram; *CBC*, complete blood count, *GI*, gastrointestinal; *ID*, infectious diseases; *IV* intravenous; *IG*, immunoglobulin; *MR*, magnetic resonance; *TSH*, thyroid stimulating hormone; *ULN*, upper limit of normal; *ICU*, intensive care unit; *DKA*, diabetic ketoacidosis; *CRP*, C-reactive protein; *ESR*, erythrocyte sedimentation rate.

**Table 3:** Cytokine release syndrome (CRS) signs and symptoms.^104^–^106^

Organ System	Patients signs and symptoms
Constitutional	Fever, fatigue, malaise, myalgias, arthralgias, anorexia
Cardiac	Tachycardia, hypotension, wide pulse pressure, cardiac dysfunction, cardiomyopathy
Dermatologic	Rash, pruritis
Pulmonary	Tachypnea, hypoxemia, dyspnea, acute respiratory distress síndrome
Gastrointestinal	Nausea/vomiting, diarrhea, transaminitis, elevated bilirubin, jaundice
Renal	Decreased urine output, azotemia, renal injury
Vascular	Hypofibrinogenemia, elevated D-dimer, coagulopathy, bleeding, disseminated intravascular coagulation
Neurologic	Headache, confusion, altered mental status, delirium, aphasia, hallucination, tremor, seizure, dysmetria, ataxia

**Table 4:** Grading cytokine release syndrome to guide treatment.^104^–^106^

Grade	Toxicity/Symptoms	Treatment
1	Symptoms are not life-threatening and include fever, nausea, fatigue, headache, myalgias	Provide symptomatic therapy, assess for infection, can continue infusion
2	Symptoms require but respond to moderate intervention: -Oxygen requirement < 40% or -Hypotension responsive to fluid or low dose of one vasopressor or -Grade 2 organ toxicity	Discontinue infusion, provide oxygen and symptomatic therapy (acetaminophen, IV fluids, NSAIDs), assess for infection
3	Symptoms require and respond to aggressive intervention: -Oxygen requirement > 40% or -Hypotension requiring high dose or multiple vasopressors or -Grade 3 organ toxicity or grade 4 transaminitis	Stop infusion, treat for infection, admit patient, provide oxygen, administer fluids and vasopressors, consider tocilizumab with steroids
4	Life-threatening symptoms: -Requirement for ventilator support or -Grade 4 organ toxicity (excluding transaminitis)	Treat as Grade 3, treat other complications (ventilator support often required)
5	Death	
